# Demographic and clinical characteristics determining patient-centeredness in endometriosis care

**DOI:** 10.1007/s00404-022-06887-5

**Published:** 2022-12-28

**Authors:** Hanna Grundström, Helena Kilander, Per Wikman, Matts Olovsson

**Affiliations:** 1 Department of Obstetrics and Gynecology in Norrköping, Linköping University, Linköping, Sweden; 2 Department of Women’s and Children’s Health, Uppsala University, Uppsala, Sweden; 3 Department of Health, Medicine and Caring Sciences, Linköping University, 581 83 Linköping, Sweden; 4 Jönköping Academy for Improvement of Health and Welfare, School of Health and Welfare, Jönköping University, Jönköping, Sweden; 5 Department of Women’s and Children’s Health, Karolinska Institutet, Stockholm, Sweden

**Keywords:** Endometriosis, Patient-centeredness, ENDOCARE questionnaire, ECQ

## Abstract

**Purpose:**

The primary aim of this study was to assess patient-centeredness of endometriosis care in a national sample of Swedish women with endometriosis. The secondary aims were to assess the importance of different dimensions of endometriosis care and to analyze demographic and clinical determinants associated with the experience of patient-centeredness.

**Methods:**

This cross-sectional study included 476 women with confirmed endometriosis. An invitation to participate was sent to 1000 randomly selected women aged ≥ 18 years having any endometriosis diagnosis and who had visited a gynecological clinic due to endometriosis problems any time during the past five years. Participants were recruited from ten different-sized gynecology clinics all over Sweden. The invitation letter had a link to the digital survey, which consisted of demographic and clinical questions, and the ENDOCARE questionnaire (ECQ). ECQ measures experiences, importance and patient-centeredness of ten dimensions of endometriosis care. Univariate and multiple regression analyses were used to analyze which patient-specific demographic and clinical determinants were associated with the experience of patient-centeredness.

**Results:**

The response rate was 48%. The results indicate that Swedish women with endometriosis experience low patient-centeredness and rate relational aspects with healthcare professionals as the most important aspects of care. Having a gynecologist with patient responsibility was an independent predictor for high patient-centeredness.

**Conclusion:**

Women with endometriosis in Sweden experience low patient-centeredness, reflecting the urgent need for improvement. More effort should be given to develop the relational aspects of care. Women with endometriosis should have a responsible gynecologist to care for treatment and follow-up.

**Supplementary Information:**

The online version contains supplementary material available at 10.1007/s00404-022-06887-5.

## What does this study add to the clinical work

**Table:** 

Overall, women were unsatisfied with the endometriosis care they had received during their lifetime, and they rated relational aspects with healthcare professionals as the most important parts of care. Endometriosis healthcare professionals should focus more on emotional support and continuity, since women having a gynecologist with patient responsibility experienced a higher patient-centeredness.	

## Introduction

Endometriosis is a chronic, inflammatory gynecological disease affecting approximately 10% of all women in reproductive age [1]. In many cases, endometriosis has a negative effect on women’s health-related quality of life (HRQoL) [2] and is associated with lower emotional, physical, psychological, social and sexual health [3, 4]. The most common symptoms are pain during menstruation and ovulation, during intercourse, urination or defecation, low back pain, and chronic pelvic pain [5].

The “gold standard” for diagnosing endometriosis is a laparoscopy with histological confirmation of endometrial tissue [5]. Typically, it takes many years to get diagnosed and to find a proper treatment [6–8]. During the road toward a diagnosis, women typically meet many different healthcare professionals. They frequently describe encounters as problematic including normalization and trivialization of symptoms [9–11].

Given the challenges with endometriosis care, there is room for quality improvements [12, 13]. There is a growing body of knowledge on the benefits of quality improvement strategies when seeking to enhance healthcare services for chronic diseases [14, 15]. The interest in improving patient-centeredness of endometriosis care has increased over the years, and improvement work for patient-centredness is today promoted at legislative and healthcare regulatory levels [16–18].

For endometriosis, patient-centeredness is defined as a combination of understanding the burden of illness and treatment from patients’ points of view while still relying on scientific knowledge [19]. In quality improvement work, we need to identify which areas of endometriosis care are of importance to women and identify patient-specific determinants associated with high patient-centeredness. This information can be used to raise healthcare professionals’ awareness to promote and preserve patient-centeredness and to tailor care on an individual level.

The primary aim of this study was to assess patient-centeredness of endometriosis care in a national sample of Swedish women with endometriosis. The secondary aims were to assess the importance of different dimensions of endometriosis care and to analyze demographic and clinical determinants associated with the experience of patient-centeredness.

## Material and methods

### Design

This cross-sectional study was conducted in a national sample of Swedish women with endometriosis recruited from ten gynecology clinics: three university hospitals, five county hospitals and two district hospitals.

### Sampling and data collection

Inclusion criteria were women aged ≥ 18 years having any endometriosis diagnosis and who had visited the clinic due to endometriosis-related problems any time during the past five years. The 150 women who had most recently visited each clinic were selected, and out of this group, 100 were randomly selected. The 1000 women were invited by mail in September 2021. A reminder was send to those who had not responded within three weeks. The invitation letter included a link to the website containing the survey.

### The digital survey

In 2011, the ENDOCARE questionnaire (ECQ) was designed to measure patient-centeredness of endometriosis care [20].

The digital survey consisted of ECQ, with the addition of three background questions: do you have a responsible gynecologist to care for your endometriosis-related problems? Do you have a plan for treatment of endometriosis? Are you currently receiving desired care for endometriosis?

The ECQ consists of 38 statements answered on a four-point Likert scale on two dimensions: experience of the statement (disagree completely, disagree, agree, and agree completely) and personal importance of the statement (not important, fairly important, important, and of the utmost importance). The statements are clustered into ten dimensions of patient-centeredness of endometriosis care: respect for patient’s values, Preferences and needs, Coordination and integration of care, Information, communication and education, Physical comfort, Emotional support and alleviation of fear and anxiety, Involvement of significant others, Continuity and transition, Access to care, Technical skills and Endometriosis clinic staff. At the end, the patient is asked to grade her overall endometriosis care on a scale from very bad (0) to excellent (10).

Three outcome measures are generated from the instrument. First, the percentage of negative experiences (PNP) is calculated on a 0 to 100 scale, with higher scores indicating worse performance. Then, the importance score (MIS) is calculated on a scale from 0 to 10, with higher scores indicating greater importance. From the PNP and MIS scores, a patient-centeredness score (PCS) is calculated and presented on a scale from 0 to 10, with higher scores indicating higher patient-centeredness [20].

The Swedish version of the ENDOCARE instrument has undergone psychometric validation and has been tested for reliability, with satisfactory results [21].

### Statistical analysis

Variables on continuous scales are described as mean and standard deviation (SD) and nominal data as frequency and percentage. To enable comparison with earlier research, MIS and PCS values are also presented as median and 25th and 75th percentiles. Missing answers were omitted in the calculations by changing the denominator in the equations for PNP, MIS and PCS. No participants had > 25% missing answers.

To analyze which patient-specific demographic and clinical determinants were associated with the experience of patient-centeredness, univariate and multiple regression analyses were used. Determinants with a *p* < 0.2 in the univariate analysis were further analyzed in a multiple regression analysis using “enter” model building in order to detect and evaluate independent predictive factors for patient-centeredness [22]. Determinants were analyzed in relation to the ten dimensions of patient-centeredness and to overall PCS. Nominal determinants with more than two categories were dichotomized.

The degree of multicollinearity was tested for the determinants in each multiple model by examining the variance inflation factor (VIF). The VIFs for these determinants were < 5, which indicates that there was no considerable multicollinearity between the variables [23].

The following determinants were analyzed: age, ever given birth (yes/no), higher education (university degree) (yes/no), currently in an intimate partner relationship (yes/no), age at first symptoms of endometriosis, patient delay (time from symptom onset to seeking care), doctor delay (time from first seeking care to diagnosis), diagnostic delay (time from symptom onset to diagnosis), number of consultations with general practitioners before referral to gynecologist, moderate/severe self-reported stage of endometriosis (yes/no), having a responsible gynecologist to care for endometriosis (yes/no), having a plan for treatment of endometriosis (yes/no), ever tried to conceive > 12 months (yes/no) and overall grading of endometriosis care.

The level of statistical significance was set at p < 0.05. Regression coefficients (β) represent the mean change in the outcome variable (PCS score) for every 1-unit of change in the determinant**,** keeping all the other determinants constant. The explained variance of the multivariate models is presented by adjusted R^2^. Data were analyzed using IBM SPSS 28.0.

## Results

In total, 476 women answered the digital survey, resulting in a response rate of 47.6%. Background characteristics and possible determinants of patient-centeredness are presented in Table 1. Participants’ mean age was 36.5 years (range 18–60). A majority had a university degree and were working full-time. Most women were currently in an intimate relationship and around half of them had children. The time between symptom onset and diagnosis (e.g., diagnostic delay) was 9.3 years. Around two out of three had a responsible gynecologist to care for endometriosis, a treatment plan and reported that they were currently receiving desired care.

**Table 1:** Study participants’ background characteristics and possible determinants of patient-centeredness (*n* = 476)

	*n* (%)*	Mean (± SD)
Age in years		36.5 ± 9
Highest level of education		
Compulsory school	27 (6)	
Secondary education	186 (39)	
University education	262 (55)	
Occupation		
Working full-time	250 (53)	
Working part-time	82 (17)	
Studying	56 (12)	
On sick leave	49 (10)	
Other	39 (8)	
Currently in an intimate partner relationship	365 (77)	
Ever tried to conceive > 12 months	163 (34)	
Has one or more child(ren)	240 (51)	
Age at first symptoms of endometriosis		19.4 ± 8
Years between first symptoms and search for help (patient’s delay)		3.3 ± 5
Years between first search for help and diagnosis (doctor’s delay)		6.5 ± 7
Years between first symptoms and diagnosis (diagnostic delay)		9.3 ± 8
Endometriosis complaints at diagnosis		
Dysmenorrhea	440 (93)	
Dyspareunia	285 (65)	
Lower abdominal pain while not menstruating	389 (85)	
Infertility	144 (34)	
Endometriosis-related symptoms during the past year		
Dysmenorrhea	273 (69)	
Dyspareunia	283 (71)	
Lower abdominal pain while not menstruating	379 (91)	
Infertility	101 (29)	
Stage of endometriosis (self-reported)		
Minimal/mild	73 (15)	
Moderate/severe	204 (73)	
Number of GP consultations before referral		6.8 ± 7
Having a responsible gynecologist to care for endometriosis	315 (66)	
Having a plan for treatment of endometriosis	301 (63)	
Currently receiving desired care	293 (62)	
Grading of endometriosis care (scale 0–10)		4.2 ± 3
Would recommend current care to others	318 (67)	

*GP* general practitioner, *SD* standard deviation

*% of valid answers

As shown in Table 2, the overall mean PCS score was 3.73, indicating a low patient-centeredness. The dimension with the highest PCS was “Endometriosis clinic staff” (mean 5.21) followed by “Respect for patients’ values, preferences and needs” (mean 5.09) and “Information, communication and education” (mean 4.81). The lowest PCS score was reported for the dimension “Emotional support and alleviation of fear and anxiety” (mean 0.85).

**Table 2:** Mean importance and patient-centeredness scores of the 10 dimensions of patient-centeredness, and overall patient-centeredness

Subscale of ECQ		Mean (± SD)	Median (25th percentile–75th percentile)
1 Respect for patients’ values, preferences and needs	MIS	9.34 (± 1.19)	10.00 (8.67–10.00)
	PCS	5.09 (± 3.72)	5.11 (2.44–8.67)
2 Coordination and integration of care	MIS	7.02 (± 2.41)	7.33 (5.33–8.67)
	PCS	3.52 (± 2.88)	3.33 (1.00–5.33)
3 Information, communication and education	MIS	8.67 (± 1.49)	9.20 (7.71–10.00)
	PCS	4.81 (± 2.81)	4.76 (2.65–7.09)
4 Physical comfort	MIS	5.85 (± 2.76)	6.00 (4.50–8.00)
	PCS	4.16 (± 2.85)	4.00 (2.25–6.00)
5 Emotional support and alleviation of fear and anxiety	MIS	7.09 (± 2.35)	7.33 (5.50–9.00)
	PCS	0.85 (± 1.54)	0.00 (0.00–1.50)
6 Involvement of significant others	MIS	6.74 (± 2.61)	7.00 (4.00–9.00)
	PCS	0.99 (± 2.09)	0.00 (0.00–1.13)
7 Continuity and transition	MIS	8.76 (± 1.62)	10.00 (7.33–10.00)
	PCS	4.61 (± 3.59)	4.22 (0.00–7.33)
8 Access to care	MIS	8.31 (± 1.74)	8.67 (7.00–10.00)
	PCS	4.48 (± 2.97)	4.20 (2.25–6.67)
9 Technical skills	MIS	9.02 (± 1.46)	10.00 (8.00–10.00)
	PCS	3.66 (± 3.23)	2.50 (0.00–6.00)
10 Endometriosis clinic staff	MIS	9.05 (± 1.39)	10.00 (8.67–10.00)
	PCS	5.21 (± 3.62)	5.33 (2.89–8.67)
Overall PCS	PCS	3.73 (± 1.94)	3.46 (2.33–5.12)

*MIS* mean importance score, *PCS* patient-centeredness score, *SD* standard deviation

The dimension “Respect for patients’ values, preferences and needs” had the highest MIS mean score (9.34), i.e., it was experienced as the most important dimension. It was followed by “Endometriosis clinic staff” (mean 9.05), “Technical skills” (mean 9.02). “Physical comfort” was experienced as the least important dimension (mean 5.85).

In the univariate regression analysis between each determinant, PCS dimensions and overall PCS, several determinants were associated with PCS (Supplement 1). Table 3 shows the results of the multiple regression analyses for each determinant having a significant and independent influence on PCS. Overall grading of endometriosis care was the determinant associated with most PCS dimensions. Having a responsible gynecologist to care for the patient was an independent determinant for the PCS dimensions “Coordination and integration of care,” “Information, communication and education,” “Emotional support and alleviation of fear and anxiety,” “Continuity and transition,” “Access to care” and for overall PCS (Table 3).

**Table 3:** Beta coefficients and 95% confidence intervals (β (95%CI)) for the determinants of patient-centeredness per PCS dimension and overall PCS in the multiple regression analyses

	PCS1: respect for patients’ values, preferences and expressed needs	PCS2: coordination and integration of care	PCS 3: information, communication and education	PCS4: Physical comfort	PCS5: emotional support and alleviation of fear and anxiety	PCS6: involvement of significant others	PCS7: continuity and transition	PCS8: access to care	PCS9: technical skills	PCS10: endometriosis clinic staff	Overall PCS
Age	–	–	–	n.s	n.s	− 0.03 (0.05 to − 0.01)	–	n.s	n.s	–	n.s
Ever given birth	–	–	–	–	n.s	–	–	–	–	–	–
Higher education	n.s	− 0.81 (− 1.41 to 0.20)	–	–	n.s	–	–	–	–	n.s	n.s
Currently in an intimate partner relationship	–	–	–	–	–	− 0.80 (− 1.33 to − 0.27)	–	n.s	–	–	–
Age at first symptoms	n.s	0.06 (0.02–0.11)	n.s	n.s	− 0.04 (− 0.06 to − 0.01)	–	–	n.s	n.s	n.s	n.s
Patient delay (years)	–	–	–	–	–	–	–	–	–	n.s	–
Doctor delay (years)	n.s	n.s	n.s	–	n.s	n.s	–	n.s	n.s	n.s	n.s
Diagnostic delay (years)	n.s	n.s	n.s	–	− 0.04 (− 0.08 to − 0.01)	n.s	–	–	n.s	n.s	n.s
No. of consultations with GPs before referral	n.s	n.s	n.s	− 0.06 (0.10 to − 0.01)	–	–	n.s	n.s	− 0.05 (− 0.10 to − 0.01)	n.s	n.s
Moderate/severe self-reported stage of endometriosis	n.s	–	–	–	–	–	–	–	n.s	n.s	n.s
Having a responsible gynecologist to care for endometriosis	n.s	0.92 (0.21–1.63)	0.71 (0.09–1.32)	n.s	0.63 (0.28–0.99)	n.s	1.78 (0.98–2.58)	1.31 (0.70–1.91)	n.s	n.s	0.61 (0.17–1.05)
Having a treatment plan	n.s	n.s	n.s	n.s	n.s	n.s	0.83 (0.42–1.62)	n.s	n.s	n.s	n.s
Ever tried to conceive > 12 months	–	–	–	–	n.s	–	–	–	–	–	–
Overall grading of endometriosis care	0.80 (0.63–0.97)	0.22 (0.10–0.34)	0.54 (0.44–0.65)	n.s	0.10 (0.04–0.16)	0.23 (0.15–0.31)	0.51 (0.38–0.64)	0.44 (0.34–0.53)	0.88 (0.75–1.02)	0.95 (0.80–1.11)	0.56 (0.49–0.64)
Model *p* value Adjusted *R*^2^	< 0.001 0.49	< 0.001 0.11	< 0.001 0.31	< 0.001 0.05	< 0.001 0.12	< 0.001 0.12	< 0.001 0.27	< 0.001 0.27	< 0.001 0.49	< 0.001 0.50	< 0.001 0.64

*n.s* not significant, *GP* general practitioner

Overall PCS had the highest explained variance (adjusted *R*^*2*^ = 0.64) and was associated with having a specific gynecologist to care for endometriosis (*β* = 0.61) and overall grading of endometriosis care (*β* = 0.56). Although the dimension “Endometriosis clinic staff” had only one significantly associated determinant, overall grading of endometriosis care (*β* = 0.95), it had a high explained variance (adjusted *R*^*2*^ = 0.50). The dimension “Physical comfort” also had only one associated determinant, numbers of consultations with GPs before referral (*β* = − 0.06), and a very low explained variance (adjusted *R*^*2*^ = 0.05) (Table 3). “Respect for patient’s values, preferences and expressed needs” had only one associated determinant, overall grading of endometriosis care (*β* = 0.80), but a relatively high explained variance (adjusted *R*^*2*^ = 0.49).

Both “Coordination and integration of care” and “Emotional support and alleviation of fear and anxiety” had four associated determinants. Three of the determinants were the same for both dimensions: age at first symptoms (β = 0.06 resp. β = − 0.04), having a responsible gynecologist to care for endometriosis (*β* = 0.92 resp. *β* = 0.63) and overall grading of endometriosis care (*β* = 0.22 resp. *β* = 0.10). However, the explained variances were relatively low for both models (adjusted *R*^*2*^ = 0.11 resp. *R*^*2*^ = 0.12) (Table 3).

Having a higher education was associated with lower scores on the dimension “Coordination and integration of care” (*β* = − 0.81), as was having an intimate partner relationship with scores on “Involvement of significant others” (*β* = − 0.80).

## Discussion

This is the first study to measure patient-centeredness and associated determinants in a larger national sample including several clinics of varying sizes.

On average, the women’s rating of overall PCS in this study was lower than what has been shown in previous comparable studies [16, 24]. An explanation could be that our data is based on a national sample including university hospitals, county hospitals and district hospitals, while earlier studies collected data from specialized endometriosis centers [16, 24].

Our results showed that “Respect for patients’ values, preferences and needs” and “Endometriosis clinic staff” were the two most patient-centered dimensions of endometriosis care, while “Emotional support and alleviation of fear and anxiety” had the lowest score. This is similar to earlier studies [16, 24]. The items measuring “Respect for patients’ values, preferences and needs” and “Endometriosis clinic staff” mainly focus on healthcare professionals’ ability to meet their patients with respect, to invite them to participate in their own care and to be supportive and friendly. The items regarding “Emotional support and alleviation of fear and anxiety” are more focused on the psychological impact of endometriosis, the opportunity to consult a counsellor and if they are given information on a patients’ organization. This could indicate that healthcare professionals being respectful and friendly is not sufficient to alleviate fear and anxiety, and more concern should be given to provide emotional support. The lack of sufficient emotional support has been highlighted before [10, 25].

The most important finding was the independent association between having a responsible gynecologist and several dimensions of PCS and overall PCS. The determinant of having a responsible gynecologist also had the highest β coefficients, meaning that it had more influence on PCS than the other determinants. Having a responsible gynecologist seems to increase the chances of experiencing patient-centeredness. In the literature, this has been described by the term “most responsible physician.” This means having a certain physician that has the responsibility for the long- and short-term medical treatment of a patient, including follow-up and evaluation [26]. According to Swedish law, clinics are obligated to provide a most responsible physician if it is necessary to satisfy a patient’s safety, continuity and coordination of care. Therefore, most patients with chronic diseases have a most responsible physician. It could be argued, that, at least, all women with complex endometriosis should have a responsible gynecologist. This is something that could be highlighted in national and international guidelines. The National Guidelines for Endometriosis Care in Sweden [27] emphasize the importance of multi-professional teams working with the more complex cases, but there are limited implications of the guidance on the continuity of care. In the recently updated endometriosis guidelines from the European Society of Human Reproduction and Embryology, there is no implication of the structure of care [28]. In our sample, two thirds had a responsible gynecologist, indicating that most clinics have a routine regarding responsible gynecologists, but the issue warrants further investigation.

Having a responsible gynecologist to care for endometriosis patients provides continuity in the contact with healthcare professionals. The importance of continuity has been noticed in endometriosis literature before [10, 11], but to the best of our knowledge, this is the first study to show an association between continuity and patient-centeredness. Apers et al. [29] showed that the ECQ dimension “Continuity and transition” was associated with overall HRQoL and the experience of emotional well-being and social support. Moreover, continuity has been identified as a specific target for improvement of patient-centeredness in endometriosis care [24, 29]. However, physicians should to bear in mind that continuity sometimes leads to a risk for tunnel vision thinking, which limits the holistic approach that is also often necessary to give proper care to women with complex endometriosis. Ideally, the care could be monitored by the responsible gynecologist in close cooperation with multiprofessional teams.

The importance of a well-functioning relationship with healthcare professionals is also reflected in the MIS scores, where “Respect for patients’ values, preferences and needs,” “Information, communication and education,” “Continuity and transition,” “Technical skills” and “Endometriosis clinic staff” were the most important dimensions. “Physical comfort” was the least important aspect, indicating that improvement work should focus on relational aspects rather than comfort in the waiting room.

Overall grading of endometriosis care was a significant determinant for overall PCS and for nine out of the ten dimensions of care. This suggests that a basic 0–10 grading scale can be used by healthcare professionals as a tool to obtain an indication of the experience of patient-centeredness in endometriosis care at their clinic. However, ECQ is preferred for a thorough assessment of patient-centeredness in endometriosis care [16].

In 2020, Schreurs et al. [30] made a secondary analysis of patient-centeredness using two studies with data from four endometriosis care centers in Belgium and the Netherlands. Their multivariate analysis showed that overall grading of endometriosis care, a lower educational level, being member of a patient organization and having seen other specialists for endometriosis complaints were independently associated with higher overall PCS [30]. Some of their results are similar to ours, where overall grading of endometriosis care gave higher PCS scores, and higher education gave lower PCS scores for the dimension “Coordination and integration of care.” The studies are not totally comparable since the included determinants vary, for example we added the background questions about having a responsible gynecologist and having a treatment plan. However, the results suggest that there might be universal factors contributing to the feelings of patient-centeredness. It would be interesting to investigate further what determinants might differ and conform between countries.

One strength of this study is that study participants constitute a random sample of women with confirmed endometriosis from ten clinics of varying sizes from different parts of Sweden, including two endometriosis specialist centers. All women had a confirmed endometriosis diagnosis, which seldom is the case in endometriosis research. Also, our population had a similar socioeconomic level as an age- and gender-matched population of Swedish women [31].

One limitation is the risk of self-selection bias, i.e., responding depends on having either very positive or negative experiences of care. Furthermore, ECQ can been criticized for risking a high recall bias, since women are obliged to answer with their lifetime endometriosis care history in mind.

The clinical implication of the results is that women with endometriosis could benefit from having a responsible gynecologist, and that clinics should organize their work around the idea of gynecologists having a handful of endometriosis patients to especially care for. Furthermore, possible interventions and actions to emotionally support women and alleviate fear and anxiety need more attention. Future studies could also focus on symptom severity and disease complexity in relation to patient-centeredness, as well as how to design team-based services together with women and healthcare professionals aiming to improve quality of care [32].

## Conclusion

In conclusion, our results show that Swedish women with endometriosis experience low patient-centeredness, reflecting an urgent need for improvement. More effort should be given to develop the relational aspects of care. Furthermore, women with endometriosis benefit from having a responsible gynecologist to care for treatment and follow-up. Given the random selection of participants from a national sample, the results should be generalizable to other countries with a similar organizational structure of healthcare.

## Supplementary Information

Below is the link to the electronic supplementary material.

Supplementary file1 (DOCX 15 KB)

## Data Availability

The participants of this study did not give written consent for their data to be shared publicly, so due to the sensitive nature of the research supporting data is not available.
