# Supplementary material for: Demographic and clinical characteristics determining patient-centeredness in endometriosis care
Source: Arch Gynecol Obstet. 2022 Dec 28;307(4):1047–55. doi: 10.1007/s00404-022-06887-5 (PMC10023645; doi:10.1007/s00404-022-06887-5)
Supplement: Supplementary file 1 — Supplementary file1 (DOCX 15 KB) [file 404_2022_6887_MOESM1_ESM.docx]

Supplement 1. *P-*values for the univariate regression analysis between each determinant and PCS dimensions, and overall PCS.

|  | **PCS1:**  **Respect for**  **patients’ values,**  **preferences and**  **expressed needs** | **PCS2:**  **Coordination**  **and**  **integration**  **of care** | **PCS 3:**  **Information,**  **communication**  **and education** | **PCS4:**  **Physical**  **comfort** | **PCS5:**  **Emotional**  **support and**  **alleviation**  **of fear**  **and anxiety** | **PCS6:**  **Involvement of**  **significant others** | **PCS7:**  **Continuity and**  **transition** | **PCS8:**  **Access**  **to care** | **PCS9:**  **Technical**  **skills** | **PCS10:**  **Endometriosis**  **clinic staff** | **Overall**  **PCS** |
| --- | --- | --- | --- | --- | --- | --- | --- | --- | --- | --- | --- |
| **Age** | 0.54 | 0.23 | 0.61 | 0.13* | <0.001* | 0.11* | 0.22 | 0.04* | 0.01* | 0.21 | 0.17* |
| **Ever given birth** | 0.29 | 0.41 | 0.85 | 0.37 | 0.03* | 0.26 | 0.56 | 0.29 | 0.58 | 0.78 | 0.49 |
| **Higher education** | 0.07* | 0.15* | 0.87 | 0.63 | 0.03* | 0.95 | 0.52 | 0.30 | 0.22 | 0.10* | 0.38 |
| **Currently in an intimate partner relationship** | 0.22 | 0.31 | 0.34 | 0.67 | 0.21 | 0.002* | 0.31 | 0.06* | 0.39 | 0.80 | 0.41 |
| **Age at first symptoms** | <0.001* | <0.001* | 0.02* | 0.02* | 0.09* | 0.84 | 0.66 | 0.09* | <0.001* | 0.12* | <0.001* |
| **Patient delay** | 0.55 | 0.78 | 0.90 | 0.63 | 0.49 | 0.42 | 0.26 | 0.21 | 0.22 | 0.03* | 0.34 |
| **Doctor delay** | <0.001* | 0.002* | 0.04* | 0.78 | 0.10* | 0.11* | 0.92 | 0.12* | <0.001* | 0.005* | <0.001* |
| **Diagnostic delay** | <0.001* | 0.004* | 0.02* | 0.66 | 0.04* | 0.03* | 0.61 | 0.44 | <0.001* | 0.18* | 0.002* |
| **No. of consultations with GPs before referral** | <0.001* | 0.005* | <0.001* | 0.003* | 0.47 | 0.72 | 0.01* | <0.001* | <0.001* | <0.001* | <0.001* |
| **Moderate/severe self-reported stage of endometriosis** | 0.16* | 0.91 | 0.24 | 0.95 | 0.60 | 0.33 | 0.57 | 0.46 | 0.13* | 0.02* | 0.13* |
| **Having a responsible gynaecologist to care for endometriosis** | <0.001* | <0.001* | <0.001* | 0.17* | <0.001* | 0.03* | <0.001* | <0.001* | <0.001* | 0.02* | <0.001* |
| **Having a treatment plan** | <0.001* | 0.02* | <0.001* | 0.007* | <0.001* | <0.001* | <0.001* | <0.001* | <0.001* | <0.001* | <0.001* |
| **Ever tried to conceive >12 months** | 0.34 | 0.94 | 0.51 | 0.53 | 0.002* | 0.27 | 0.87 | 0.67 | 0.48 | 0.21 | 0.28 |
| **Overall grading of endometriosis care** | <0.001* | <0.001* | <0.001* | <0.001* | <0.001* | <0.001* | <0.001* | <0.001* | <0.001* | <0.001* | <0.001* |

*= *p* <0.2 PCS=patient-centredness score GP=general practitioner
